# Epidemiology of generalised joint laxity in 14 year old children from the UK: a population-based evaluation

**DOI:** 10.1186/1546-0096-10-S1-A107

**Published:** 2012-07-13

**Authors:** Jacqui Clinch, Kevin Deere, Tobias John, Emma Clarke

**Affiliations:** 1 Bristol Royal Hospital for Children, Bristol, UK; 2 University of Bristol, Bristol, UK

## Purpose

Although diagnostic criteria for generalised joint laxity in children are widely used, they may have limited validity as robust descriptive epidemiology of this condition is lacking.Therefore, we used a large population-based birth cohort to describe the point prevalence and pattern of generalised joint laxity in children aged 14 years old from Bristol and the surrounding area in the South West UK.

## Methods

We performed a cross-sectional analysis of the Avon Longitudinal Study of Parents and Children (ALSPAC). Hypermobility was measured at aged 14 years using the Beighton scoring system.At the same research clinic height and weight were measured. Objective measures of physical activity were collected by accelerometry. Data were also collected on other variables such as puberty and socio-economic status by self-completion questionnaire. Simple prevalence was calculated. Chi-squared tests and logistic regression were used to assess associations between variables and the presence or absence of generalised joint laxity.

## Results

6022 children were evaluated. 45% of girls and 29% of boys had hypermobile fingers. Using a cut-off of ≥4, the prevalence of generalised joint laxity in girls and boys aged 13.8 years was 27.5% and 10.6% respectively. When using different definitions of generalised joint laxity (≥4 or ≥6) there were no strong consistent associations seen. However, there was a suggestion of a positive association between generalised joint laxity in girls and physical activity (OR 2.87, 95%CI 1.04 to 7.91 for more than 60 minutes of moderate or vigorous activity compared to less than 60 mins), body mass index (OR2.70, 95%CI 1.245 to 5.88 in obese compared to underweight) and maternal education (OR3.13, 95%CI 1.18 to 8.36 in mothers educated to degree level compared to those with no formal qualifications). No associations were seen in boys.

## Conclusion

We have shown for the first time that the prevalence of generalised joint laxity in the general population of children aged 14 years is 27.5% in girls and 10.6% in boys when using the standard definition of ≥4 hypermobile joints, suggesting this cut-off is too low. We have also shown a suggestion of positive associations between generalised joint laxity in girls and physical activity, BMI and maternal education. These results give a platform to evaluate the relationships between the Beighton criteria and features such as pain, thereby allowing evaluation of the clinical validity of the score

## Disclosure

Jacqui Clinch: None; Kevin Deere: None; Tobias John: None; Emma Clarke: None.

